# Prescription Department

**Published:** 1894-10

**Authors:** 


					﻿Prescription Department.
CHRONIC RHEUMATISM.
By Prof. J. Solis Cohen, Philadelphia.
R Sodiisalicylatis, 3 iv.
Tinct. ferri chloridi, f 3 iv.
Acidi citrici, grs. x.
Glycerini, f 5 iss.
01. gaultherise, m, viij.
Liq. ammon. Citratis, q. s. ad.
f 5 iv. M.
Sol. sec. art. Dos f 5 i or f 5 ij.
Dissolve the citric acid and sodium
salicylate in the liquor ammonise-
citratas. To the glycerin add the
tincture of chloride of iron, and then
mix the two solutions.
TO ALLAY THE CRAVING FOR ALCO’Scm.
R Tin?, capsici.
Tine, nucis vom.
Acidi nitro-hydrochloric, dilute,
aa 3 j.
Infus. gentian, ad. 5 xii.
M. and make mixture. Sig. Two
tablespoonfuls as often as required.
CHOLERA INFANTUM.
R Bismuth, sub. nit., 5 ss.
Tr. opii.. gtt. xx.
Syr. ipecac,	) --	...
Syr. rhei, arom., J ® J»
Listerine, 3 ss.
Mist, cretee, 5 j. M.
Sig. Teaspoonful as often as nec-
essary, birt not more frequently than
every three or four hours. This for
children about ten or twelve months
old.
ECZEMA OF SCALP.
Eczema of scalp in adults is treated
by Dr. H. G. Piffard with the follow-
ing mixture:
R Acidi salicylici, gr. xv.
Olei pini sylvestris, § ii.
Olei ricini, 3 iss.
Olei lavandulse, 3 iiiss.
Olei citronellse, 3 ss.
M. Sig. Apply once daily by rub-
bing a few drops with the finger into
the affected part.
EXCORIATION TN CHILDREN.
R Salicylic acid, 5 dgms. (grs. viij.).
Subnitrate of bismuth, 8 gms.
(3 ij)-
Starch, 6 gms. (5 jss.)
Rose ointment, 30 gms. (3 j).
For external use.
DIURETIC IN CARDIAC AND RENAL
DROPSY.
R Fl. ext. jalap.
Fl. ext. squills, aa 15 gms. (3 iv).
Fl. ext. jaborandi, 30 gms. (3 j).
Fl. ext. digitalis, gtt. xxx.
Nitrate of potash, 29 gms. (3 v).
Angelica wine, 60 gms. (3 ij).
A tablespoonful every three hours.
PERIOSTITIS.
R Hydrag. biniodide, gr. x.
Potass, iodidi, 3 j.
Ext. belladonnae, 9 j.
Ungt. cerci, § ij.
M. f. ungt.
Sig. Apply externally.
FETID DIARRHEA IN CHILDREN.
By Dr. E. Tompkin.
R Calomel, gr. jss.
Sulphocarbolate of zinc, 3 ijss.
Bismuth subnitrate, 3 ij.
Pepsin, grs. xxx.
Divided into twelve powders. Three
powders daily in a child of one year.
WHOOPING COUGH.
By Prof. Graham.
R Ammonii bromidi, 3 iij-
Ammonii carbonat., gr. j.
• Syrup tolu, f £ j.
Aquae dest. q. s. ad. f 3 iij. M.
Sig. Teaspoonful every four hours.
DIPSOMANIA.
By Dr. Edward. C. Mann.
R Quininae sulph., gr. ij.
Zinci oxid., gr. ij.
Stryehninae sulph., gr. 1-40.
Acid arseniosi, gr. 1-100.
Pulv. capsici, gr. ij.
M. et ft. pil. No. j. Sig. One pill
thrice daily.
				

